# Selection of optimal reference genes for qRT-PCR analysis of shoot development and graviresponse in prostrate and erect chrysanthemums

**DOI:** 10.1371/journal.pone.0225241

**Published:** 2019-11-27

**Authors:** Xiaowei Li, Yujie Yang, Sagheer Ahmad, Ming Sun, Cunquan Yuan, Tangchun Zheng, Yu Han, Tangren Cheng, Jia Wang, Qixiang Zhang

**Affiliations:** Beijing Advanced Innovation Center for Tree Breeding by Molecular Design, Beijing Key Laboratory of Ornamental Plants Germplasm Innovation & Molecular Breeding, National Engineering Research Center for Floriculture, Beijing Laboratory of Urban and Rural Ecological Environment, Engineering Research Center of Landscape Environment of Ministry of Education, Key Laboratory of Genetics and Breeding in Forest Trees and Ornamental Plants of Ministry of Education, School of Landscape Architecture, Beijing Forestry University, Beijing, China; Nazarbayev University, KAZAKHSTAN

## Abstract

The prostrate cultivars of ground-cover chrysanthemum have been used in landscape gardening due to their small stature, large crown width and strong branching ability. qRT-PCR is a rapid and powerful tool for gene expression analysis, while its accuracy highly depends on the stability of reference genes. The paucity of authentic reference genes presents a major hurdle in understanding the genetic regulators of prostrate architecture. Therefore, in order to reveal the regulatory mechanism of prostrate growth of chrysanthemum stems, here, stable reference genes were selected for expression analysis of key genes involved in shoot development and graviresponse. Based on transcriptome data, eleven reference genes with relatively stable expression were identified as the candidate reference genes. After the comprehensive analysis of the stability of these reference genes with four programs (geNorm, NormFinder, BestKeeper and RefFinder), we found that *TIP41* was the most stable reference gene in all of the samples. *SAND* was determined as a superior reference gene in different genotypes and during the process of shoot development. The optimal reference gene for gravitropic response was *PP2A-1*. In addition, the expression patterns of *LA1* and *PIN1* further verified the reliability of the screened reference genes. These results can provide more accurate and reliable qRT-PCR normalization for future studies on the expression patterns of genes regulating plant architecture of chrysanthemums.

## Introduction

Quantitative real-time reverse-transcription polymerase chain reaction (qRT-PCR), a powerful tool for analyzing gene expression profiles, is commonly used in many research fields because of its high sensitivity, accuracy, specificity, throughput and low cost [1,2]. However, the accuracy of qRT-PCR analysis is highly dependent on an appropriate choice of reference genes [3]. The use of improper reference genes can lead to conflicting expression data [4,5]. The expression level of optimal reference genes for specific experimental system must be stable both in time and space, and should be unaffected by any treatment or genetic manipulation [6]. Whereas there are no universal reference genes that are stably expressed in all of the tissues and under all conditions [7]. *EF1α* (*elongation factor 1α*) was stably expressed during aphid infestation but with unstable expression during waterlogging stress, and *PP2A* (*protein phosphatase 2A*) was stably expressed under heat and waterlogging stress but the least stable reference gene for aphid infested plants [8]. *MTP* (*metalloprotease*) and *ACT* (*actin*) were the most stable in diploid and tetraploid *Chrysanthemum nankingense*, while *PSAA* (*photosynthesis-related plastid gene representing photosystem I*) and *EF1α* were the most stable in tetraploid and hexaploid *C*. *zawadskii* [6]. *SAND* (*SAND family protein*) was the most stable reference gene in floral developmental process in *C*. *lavandulifolium* [9]. Therefore, it is essential to earnestly evaluate and validate the stability of reference genes for every specific experimental design before expression analysis of target genes by qRT-PCR experiments.

Chrysanthemum has long been cultivated worldwide as a cut flower, in the garden and as a potted flower owing to its rich germplasm among ornamental plants [10–13]. Ground-cover chrysanthemum, a cultivar group of *Chrysanthemum morifolium*, is widely used in landscape gardening due to its large canopy, strong branching ability, large numbers of capitula, strong resistance and wide adaptability [14,15]. Cultivars of ground-cover chrysanthemum with vertical architecture are common, while the creeping or prostrate type is rare. If the prostrate architecture of *C*. *yantaiense* (abbreviated as YT), an outstanding trait with application value in landscape gardening, is introduced into ground-cover chrysanthemums, the ground-covered ability will be increased and greening costs will be reduced [16]. Most mutants with prostrate habit in model plants have been identified to be related to the loss of gravitropism or reduced gravitropism of the above-ground part. During graviresponse, *UBQ* (*Ubiquitin*) and *ACT* were primarily used as reference genes. *UBQ* was used for data normalization in qPCR analysis after gravity stimulation in Arabidopsis inflorescence stems [17] and rice shoot base [18,19]. *ACT* was used as an internal control to analyze relative expression level under graviresponse in peanut gynophores [20]. In the case of *chrysanthemum*, however, previous researchers have mainly focused on anatomical physiological characteristics of the creeping stem [21,22]. Few studies have reported expression patterns of gravitropic-related genes in chrysanthemum. When analyzing the expression pattern of gravitropic-related genes between upper and lower side of the creeping stem after gravistimulation, *ACT* was used as the housekeeping gene in chrysanthemum cultivar ‘Yuhuajinhua’ [23]. On account of no systematic evaluation of reference genes during shoot development and gravitropic response in chrysanthemum, it is imperative to identify stable reference genes before analyzing expression patterns of key genes involved in shoot development and graviresponse in prostrate and erect chrysanthemum.

In the present study, eleven reference genes, including four conventional reference genes (*ACT*, *EF1α*, *GAPDH*, and *UBQ*) and seven new ones (*PP2A-1*, *PP2A-2*, *SAND*, *TIP41*, *PGK*, *MTP*, and *SKIP16*), were selected based on model plants and chrysanthemum transcriptome libraries. The stability of these reference genes in shoot development and gravitropic response was then evaluated by geNorm, NormFinder, BestKeeper and RefFinder. So as to validate the effectiveness of the screened reference genes, the expression patterns of *LA1* and *PIN1* were analyzed. This study provides more accurate and reliable qRT-PCR normalization for future researches on expression patterns of genes regulating plant architecture of chrysanthemums.

## Materials and methods

### Plant materials

**Fig 1:**
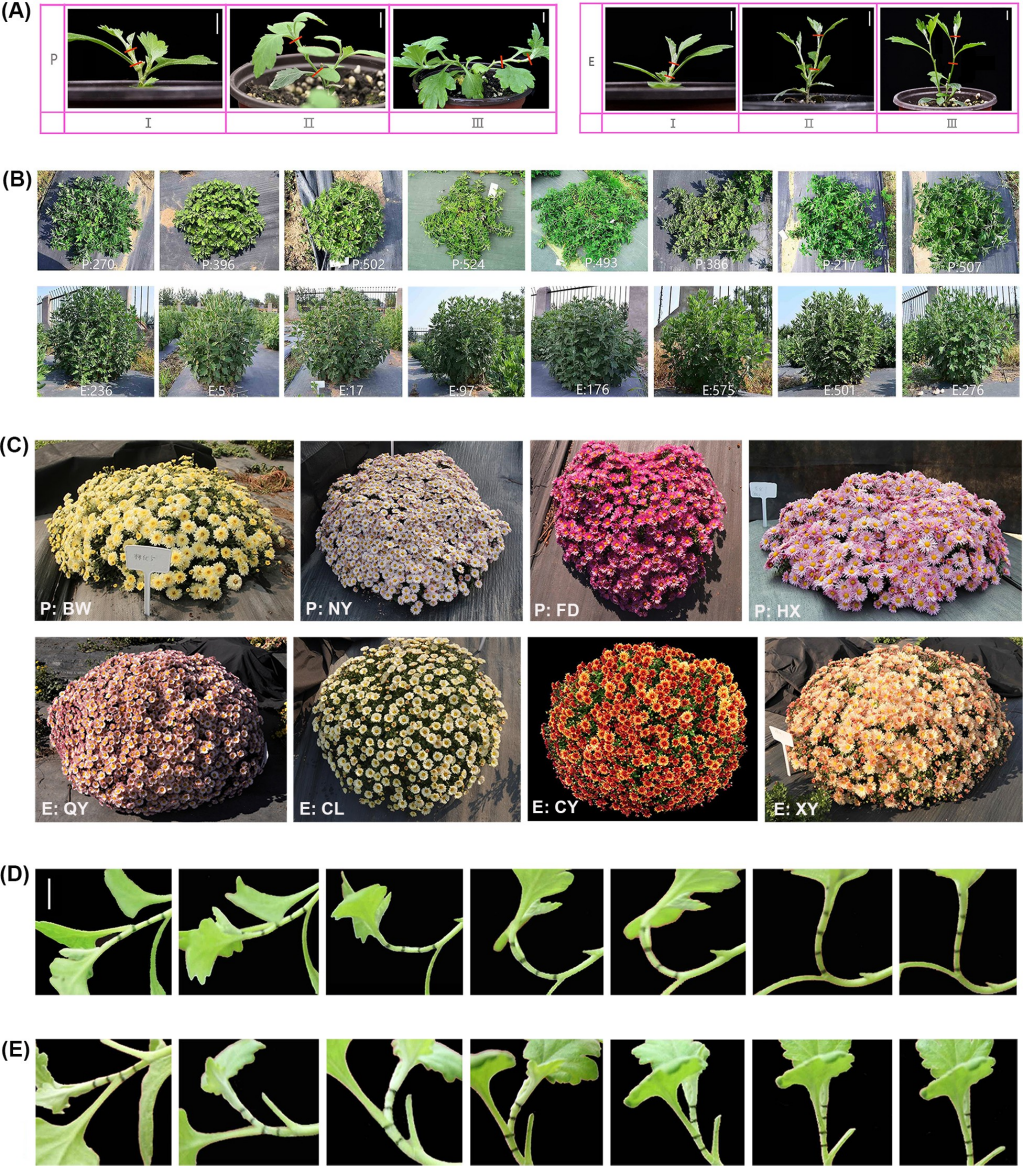
Different developmental stages, genotypes and gravitropic responses of prostrate and erect chrysanthemums. (A) Set 1: shoots of prostrate and erect bulk at three developmental stages. (**B**) Set 2: sixteen prostrate and erect strains. (**C**) Set 3: eight new cultivars of ground-cover chrysanthemum. (**D**) Set 4: gravitropic responses of stems of YT at different time points (0, 3, 6, 9, 12, 24, and 48 h). (**E**) Set 5: gravitropic responses of stems of FH at different time points (0, 3, 6, 9, 12, 24, and 48 h). I, Stage I; II, Stage II; III, Stage III; P, prostrate; E, erect; BW, ‘Fukanbowu’, CNA20170990; NY, ‘Fukannongyun’, CNA20170985; FD, ‘Fukanfendai’, CNA20170984; HX, ‘Fukanhongxiu’, CNA20170986; QY, ‘Beilinqiuyun’, CNA20170987; CL, ‘Fukanchenlu’, CNA20170988; CY, ‘Fukanchiyan’, CNA20170989; XY, ‘Fukanxiaoyue’, CNA20170983.

YT displays a prostrate growth habit with a height of less than 20 cm and the stem GSA (gravitropic setpoint angle) value of 90°–100°. While, ‘Fanhuasijin’ (CNA20090874, abbreviated to FH), a cultivar of ground-cover chrysanthemum, shows a vertical architecture with high stature of 60–70 cm and the stem GSA of 170°–180°. F_1_ and BC_1_ populations segregating for prostrate and erect architecture were constructed by crossing YT and FH. Sixteen F_1_ progenies with prostrate growth habit were selected to construct the ‘prostrate’ bulk and 16 F_1_ progenies with erect architecture were chosen to generate the ‘erect’ bulk. Uniformly-sized cuttings from each selected strain were planted into a 1:1 mixture of vermiculite and perlite. Rooted cuttings were then transplanted into individual pots (10*8.5*9.5 cm) with a 1:1 mixture of peat and perlite. To achieve the great consistency and obtain the gene expression profile of the early stage, the terminal buds were removed uniformly two weeks after transplanting. Based on the morphologic changes of the prostrate bulk from preliminary experiment, the consecutively developmental process of stem was divided into three stages: Stage I (1 week after cutting the terminal bud, GSA of stem from prostrate bulk was 150°–180°), Stage II (3 weeks after cutting the terminal bud, GSA of stem from prostrate bulk was 105°–150°) and Stage III (5 weeks after cutting the terminal bud, GSA of stem from prostrate bulk was 90°–105°). Thus, the stems of prostrate and erect bulk at three stages were collected as set 1 in this study (Fig 1A). Set 2 consisted of 16 prostrate and erect strains at Stage III (Fig 1B). Set 3 was composed of eight new cultivars of ground-cover chrysanthemum from BC_1_ population, including four prostate cultivars (‘Fukanbowu’ ‘Fukannongyun’ ‘Fukanfendai’ and ‘Fukanhongxiu’) and four vertical cultivars (‘Beilinqiuyun’ ‘Fukanchenlu’ ‘Fukanchiyan’ and ‘Fukanxiaoyue’) at Stage III (Fig 1C). The cutting seedlings were maintained in a greenhouse under long-day photoperiod (16 h light/8 h dark), the day/night temperature of 25/18°C, and the relative humidity of 70%.

Cutting-seedlings of YT and FH at the 10–12 leaf stage were subjected to gravistimulation by gently rotating the pot 90° in a growth chamber in the dark with the temperature of 22±1°C and the relative humidity of 60% [18]. Stems of FH displayed obviously asymmetric growth at 3 h after horizontal treatment and regained vertical growth in less than 12 h. However, shoots of YT began to bend at 6 h and it took more than 24 h for YT to completely recover upright growth. Segments from the curved internode were separately collected at 0, 1, 3, 6, 9, 12, 24, and 48 h. Then, these stem segments were rapidly divided into the upper and lower half by cutting along the mid longitudinal axis of the stem [23]. Dissected segments from YT and FH at eight time points composed set 4 (Fig 1D) and set 5 (Fig 1E), respectively. All of the samples were collected in triplicates, immediately frozen in liquid nitrogen and then stored at −80°C.

### RNA extraction, DNase I digestion and first-strand cDNA synthesis

Total RNA from all samples was isolated using E.Z.N.A Plant RNA Kit (OMEGA BIO-TEK, Norcross, USA) according to the manufacturer’s instruction. Purity and concentration of total RNA were detected by a UV-Vis Spectrophotometer Q5000 (Quawell, San Jose, USA). The RNA samples showing an A_260_/A_280_ ratio of 1.9−2.2 and an A_260_/A_230_ ratio greater than 2.0 were used for subsequent experiments. The integrity of RNA was further assessed by 1% (w/v) agarose gel electrophoresis. RNA samples with a 28S/18S ratio of 1.5–2.0 and without smears were used for further analysis. 1.0 μg total RNA was reverse-transcribed by the FastQuant RT Kit (with gDNase) (TIANGEN, Beijing, China) according to operating manual. All of the synthesized cDNAs were stored at −20°C until PCR experiments.

### Selection of candidate reference genes and primer designing

The candidate reference genes were derived from model plants and related species. Those nucleotide sequences were used as query sequences to search the *Chrysanthemum morifolium* transcriptome libraries (NCBI SRA accession: SRP173747) using the TBLASTX program. Several sequences with high similarity (e-value < 10^−9^) were obtained. From these sequences, a number of sequences stably expressed (|log2Ratio| < 0.3) were screened as candidates. The primers of those candidates were designed using Primer Premier 5.0 and shown in S2 Table. PCR products of reference genes were purified and then cloned into pMD18-T vector (TaKaRa, Japan). The positive clones were sequenced by Sangon Biotech Co. Ltd. (Sangon, China). Specific primers of candidate reference genes for qRT-PCR were designed using PrimerQuest® tool (https://sg.idtdna.com/Primerquest/Home/Index) with primer Tm of 55−65°C, primer length of 19−25 bp and short PCR products of 100−250 bp.

### Test of specificity and amplification efficiency of primers

The performance of the primers was evaluated by 1% (w/v) agarose gel electrophoresis and qRT-PCR. Each amplicon was purified using TIANgel Midi Purification Kit (TIANGEN, China) and cloned into pMD18-T vector (TaKaRa, Japan). The positive clones were sequenced by Sangon Biotech Co. Ltd. (Sangon, China). The specificity of each primer pair was accepted when it conform to the following criteria: (1) Only a specific product was generated for cDNA template, (2) no product was generated for genomic DNA template, and (3) the melting curve of qRT-PCR showed a single peak. Amplification efficiency (E) and correlation coefficient (R^2^) of each primer pair were determined by the slope of a standard curve generated from serial dilutions (×1, ×5, ×25, and ×125) of pooled cDNA samples as the template [2].

### qRT-PCR assays

qRT-PCR experiments were conducted using the SYBR *Premix Ex Taq* II Kit (TaKaRa, Japan) based on a PikoReal Real-time PCR system (Thermo Fisher Scientific, USA). Each reaction was prepared in a total volume of 20.0 μl containing 2.0 μl of diluted cDNA (~20 ng), 0.8 μl of each primer (10 μM, Sangon Biotech, Shanghai), 10.0 μl of 2 × SYBR *Premix Ex Taq* II, and 6.4 μl of double-distilled water. The amplification in all reactions was performed according to following program: 95°C for 30 s, 40 cycles of 95°C for 5 s and 62°C for 30 s, 72°C for 30 s. Melting curve was recorded at the end of the qPCR by heating from 60°C to 95°C with 0.2°C increment every 1 s. Negative controls with no-RT RNA were conducted to ensure there was no genomic contamination. No template controls were also conducted with double-distilled water as template. Each reaction was carried out with three technical replicates.

### Stability analysis of reference genes

The expression levels of the candidate reference genes in all of the samples were determined by quantification cycle (Cq) values. Box plots of raw Cq values were generated using SPSS v22.0 software (IBM, Chicago, IL, USA) to exhibit variation of each reference gene in all tested samples.

The expression stability of 11 candidate reference genes was assessed using four computational programs, including geNorm [24], NormFinder [25], BestKeeper [26] and RefFinder (http://150.216.56.64/referencegene.php). For geNorm and NormFinder analysis, Cq values were converted into relative quantities by the 2^-ΔCq^ method, in which ΔCq = each corresponding Cq value—minimum Cq value [27]. The geNorm program calculates stability measure (M) of gene expression based on the average pairwise variations (V) of a particular gene against all other reference genes. The lowest M value represents the most stable expression, and M value < 0.5 is taken as an indicator of stable expression [28]. NormFinder program (V0953, Aarhus, Denmark) calculates intra- and inter-group variations, and then combines the two results into a stability value of each candidate gene. Genes with lowest stability value are the most stable. BestKeeper (Version1.0, Munich, Germany) ranks the expression stability of reference genes according to the coefficient of variance (CV) and the standard deviation (SD) of the Cq values, and the reference gene with lowest CV and SD is identified as the most stable one. RefFinder is an online tool that integrates four methods (geNorm, NormFinder, BestKeeper, and ΔCT) to compare and rank the stability of candidate reference genes comprehensively.

### Validation of selected reference genes

*LAZY1* (*LA1*) is the principal determinant of branch angle and mediates plant architecture [29,30,18,31–34]. *PIN1*, one of the *PIN-FORMED* (*PIN*) family members, encodes an auxin efflux carrier involved in the auxin redistribution in gravitropic response [35,36]. The primers of *LA1* and *PIN1* for qRT-PCR are presented in Table 1. The transcription of these genes was quantified using both stable and unstable reference genes to validate the reliability of the results. The expression levels of these target genes were calculated using the 2^-ΔΔCq^ method [27].

**Table 1:** Primer sequences and amplicon characteristics of target genes.

Gene symbol	GenBank ID	Primer sequence (5’–3’) Forward/Reverse	PCR efficiency (%)	Regression coefficient (R^2^)	Amplicon length (bp)	Melting TM (°C)
*LA1*	MK381414	GCAACATTCCACAGGCTACA/	90.1	0.995	95	82.7
		CAGCTCCAACACCAGGTAATC				
*PIN1*	MK381416	GAAGTGGTGTGAGTCCAAGAA/	106.3	0.996	95	79.8
		CCCTCCTTGACTACCATTAACC				

## Results

### Identification of candidate reference genes from transcriptome libraries

Based on previous studies on selection of reference genes in Arabidopsis [3,37] and chrysanthemums [6,8,9,38,39], fifteen reference genes were ubiquitously expressed in six different transcriptome libraries of *C*. *morifolium* and passed the BLAST test. Eleven of these genes, including *PGK* (*phosphoglycerate kinase*), *MTP* (*metalloprotease*), *PP2A-1* (*protein phosphatase 2A-1*), *PP2A-2* (*protein phosphatase 2A-2*), *ACT* (*actin*), *EF1α* (*elongation factor 1α*), *GAPDH* (*glyceraldehyde-3-phosphate dehydrogenase*), *TIP41* (*TIP41-like family protein*), *UBQ* (*ubiquitin extension protein*), *SAND* (*SAND family protein*) and *SKIP16* (*SKP1/ASK-interacting protein 16*), showed the most stable expression with |log2Ratio| < 0.3 (S1 Table). Therefore, they were selected as candidates for normalizing the gene expression during shoot development and gravitropic response. Moreover, all candidate reference genes were cloned and sequenced. The corresponding NCBI accession numbers are given in Table 2.

**Table 2:** Primer sequences and amplicon characteristics of candidate reference genes.

Gene symbol	GenBank ID	Primer sequence (5′–3′) for qPCR Forward/Reverse	PCR efficiency (%)	Regression coefficient (R^2^)	Amplicon length (bp)	Melting TM (°C)
*PGK*	MK381403	CGTTGGTTATTCTTGTATGTGGC/	102.8	0.995	175	83.2
		CTGAAGTCTCGTGCCCATATAG				
*MTP*	MK381404	GATTAAAGCCAACAGTCTTGCG/	94.3	0.999	158	84.8
		ACGTTCCAAGTATCTCAATCCTG				
*PP2A-1*	MK381405	TTGGCGGATATGGTGATTAGG/	96.6	0.997	109	79.9
		GTTGTGTTGCTTCAAGAACCTC				
*PP2A-2*	MK381406	ATCAGAACAGGAGGTCAGG/	102.9	0.997	171	82.8
		TAATTTGTATCGGGGCACTT				
*ACT*	MK381407	AGCCGTTCTTTCCCTGTATG/	106.9	0.997	186	83.8
		GAATACCCACGCTCTGTAAGG				
*EF1α*	MK381413	CAATTGCTAAACCATCTGCCG/	103.8	0.994	230	82.0
		AGGCTTGAACTGTGAACGAG				
*GAPDH*	MK381408	CCCACCTTCTCAAATACGACTC/	98.4	0.999	175	84.4
		ACTCCTGTCCCTTCTATCACC				
*TIP41*	MK381409	TGAGTTGGCTGATAATGGAGTC/	98.0	0.999	172	82.1
		TCGGACTATAACAGGCTTTGC				
*UBQ*	MK381410	ATCTTGTGTTGAGGTTGAGGG/	105.4	0.999	246	83.3
		GGAGACGAAGGACAAGATGAAG				
*SAND*	MK381411	TTACCTGTTGACCCATCTGC/	106.6	0.994	135	82.3
		CATAAAGTGCCAAAGTCCAGC				
*SKIP16*	MK381412	AGCTGTCTGAACCTGTTGATC/	98.9	0.997	139	82.2
		CTTCTGATTCTGTCCCAAACG				

### Performance of primers for each reference gene

**Fig 2:**
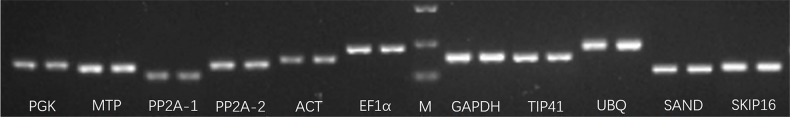
Agarose gel electrophoresis of amplicons of eleven candidate reference genes. Marker (M) from top to bottom: 500, 250 and 100 bp. The primer pairs in each separation are ordered from left to right as *PGK*, *MTP*, *PP2A-1*, *PP2A-2*, *ACT*, *EF1α*, *GAPDH*, *TIP41*, *UBQ*, *SAND*, *SKIP16* (two technical replicates).

The primer sequences and amplicon length of eleven reference genes are presented in Table 2. The performance of the primers was evaluated by agarose gel electrophoresis and qRT-PCR. The agarose gel electrophoresis results showed that all eleven primer pairs amplified a single product of expected size (Fig 2). The sequences of amplicons were almost identical (similarity of 98%–100%) to that of transcriptome data of *C*. *morifolium*. Melting curve analysis was performed by qRT-PCR after 40 cycles of amplification, and the presence of a single peak in melting curve further confirmed the specificity of all tested primer pairs (S1 Fig). The amplification efficiencies of these primers ranged from 94.3% to 106.9% and the linear standard curve for each gene from a five-fold serial dilution of cDNA showed R^2^ > 0.99 (Table 2 and S2 Fig). These results reflected the high specificity and quality of the qRT-PCR, thus these eleven pairs of primer of candidate genes were used in the further assay.

### Expression profiles of candidate reference genes

**Fig 3:**
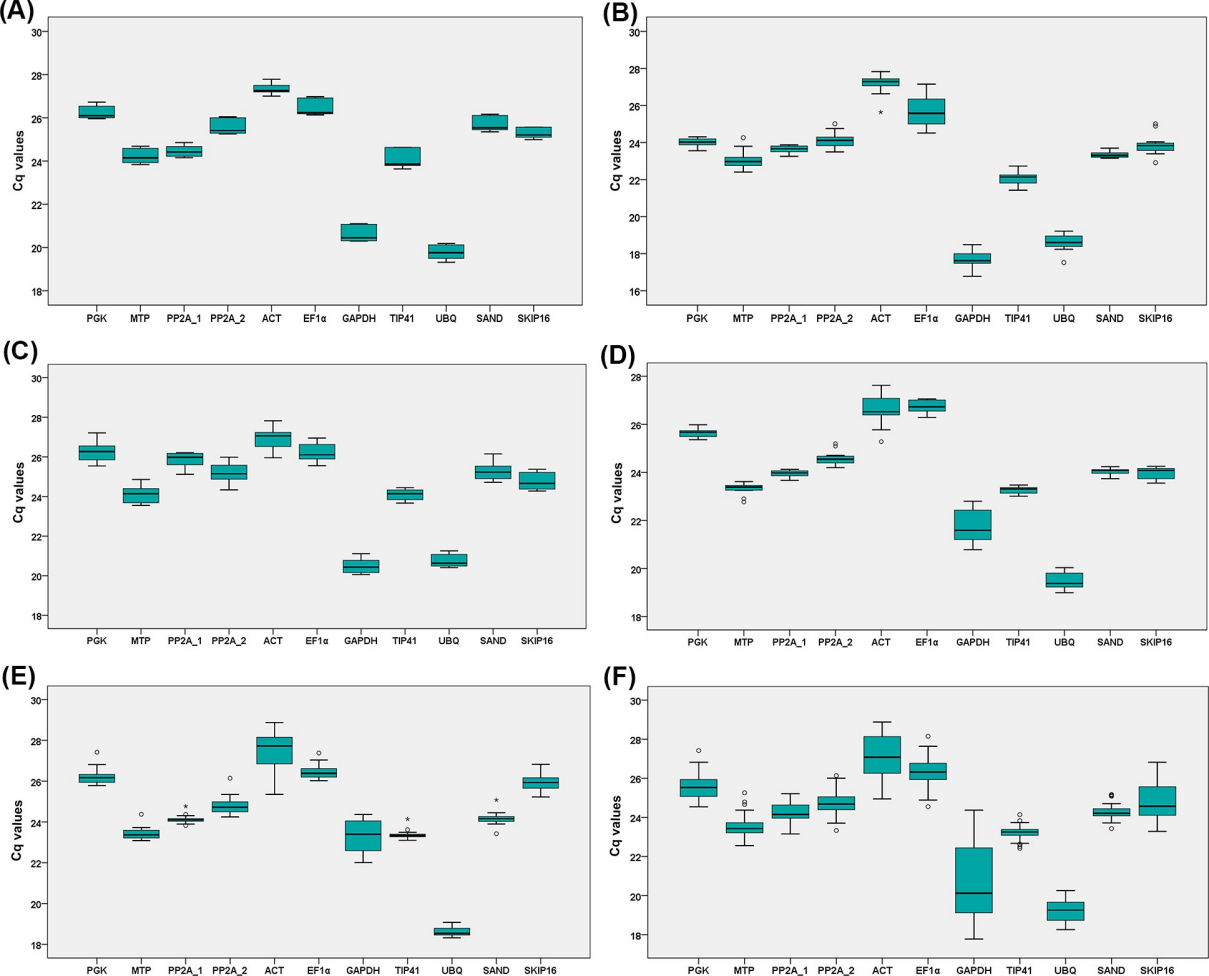
The Cq values of the eleven candidate reference genes depicted with boxplots. (**A**) Set 1: stems of ‘prostrate’ and ‘erect’ bulk at three stages. (**B**) Set 2: stems of sixteen prostrate and erect strains at Stage III. (**C**) Set 3: stems of eight new cultivars of ground-cover chrysanthemum. (**D**) Set 4: gravitropic responses of stems of YT at different time points (0, 1, 3, 6, 9, 12, 24, and 48 h). (**E**) Set 5: gravitropic responses of stems of FH at different time points (0, 1, 3, 6, 9, 12, 24, and 48 h). (**F**) The Cq values of the eleven candidate reference genes across all samples. The upper and lower edges of the box represent the upper and lower quartiles, and the middle black line is the median. The upper and lower whiskers depict the smallest and largest unbooked values. Circles and asterisks indicate mild and extreme outliers, respectively.

Quantification cycle (Cq) values of candidate reference genes are shown in boxplots (Fig 3). The Cq values ranged from 17.77 to 28.88 across all samples. Among the eleven candidate reference genes, *ACT* showed the lowest expression level with a mean Cq value of 27.13, while *UBQ* displayed the highest expression level with a mean Cq value of 19.25 (Fig 3F). The raw Cq values of candidate reference genes fluctuated in varying degrees in each set and across all samples, indicating that none of these genes had a constant expression level (Fig 3A–3F). Preliminary analysis of raw Cq values with boxplots could not provide enough information on expression stability. Thus, four computational programs were used to further evaluate the stability of reference genes for chrysanthemum in different shoot development stages and gravitropic response.

### The stability of candidate reference genes

We analyzed the stability of candidate reference genes among each set individually and the overall stability among total samples. Four computational programs, including geNorm, NormFinder, BestKeeper, and RefFinder, were used to evaluate the stability of candidate reference genes.

#### geNorm analysis

**Fig 4:**
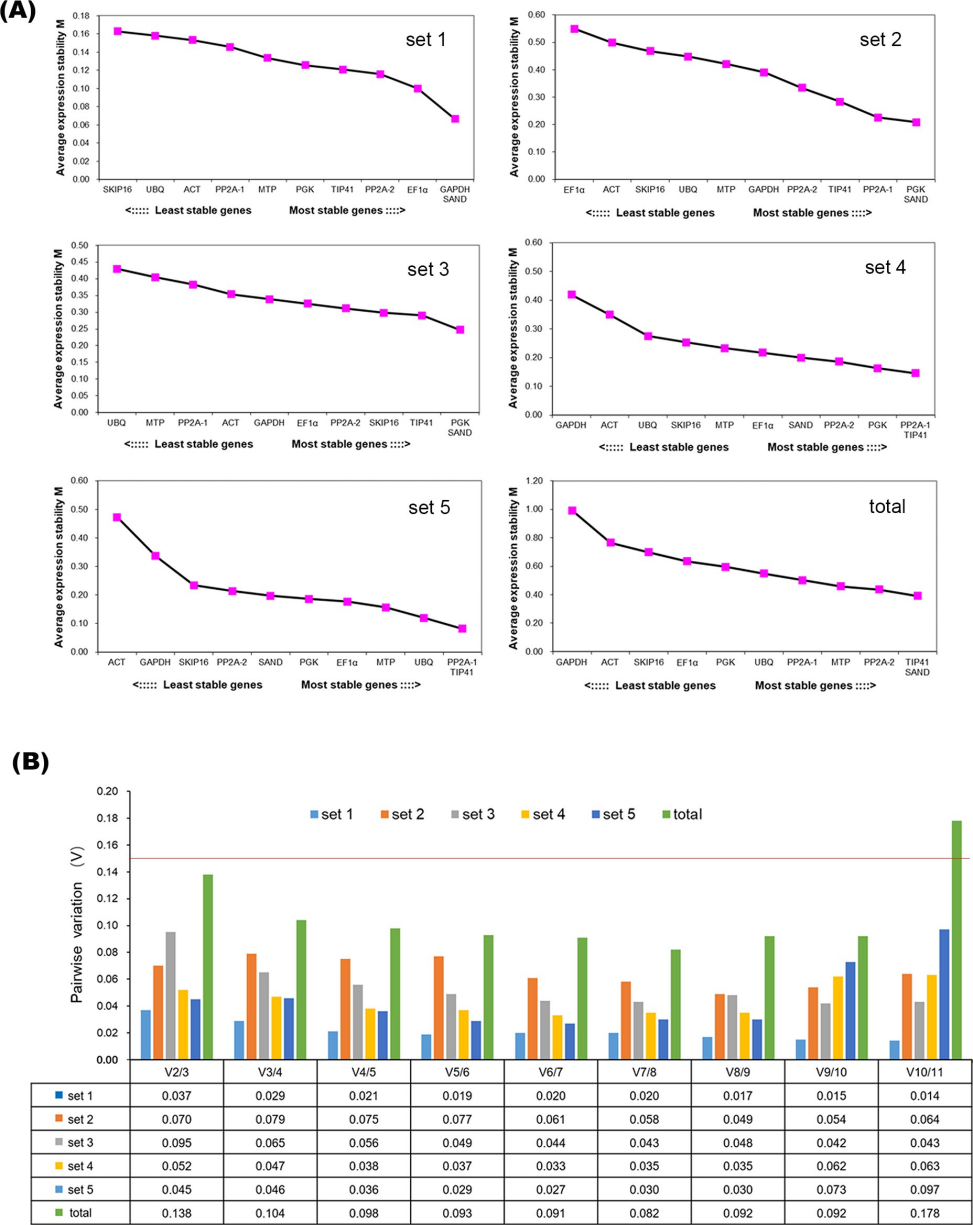
Average expression stability (M) and pairwise variation (V) of the candidate reference genes calculated by geNorm. (**A**) Average expression stability values M after stepwise exclusion of the least stable genes across all sets. (**B**) Determination of optimal number of reference genes required for accurate normalization.

The geNorm program was used to rank the expression stability of candidate reference genes by calculating the average expression stability (M) [24]. A lower M value indicates a more stable gene expression, and the M value should be lower than 0.5 [28]. The ranking orders of each set based on the M value are depicted in Fig 4A. Most genes were stable with M value below 0.5, except for *EF1α* (M = 0.549) in set 2. In set 1, *SAND* and *GAPDH* were the most stable genes with the lowest M value of 0.067, while *SKIP16* was the least stable one with the highest M value of 0.164. The most stably expressed genes in set 2 were *SAND* and *PGK* with an M value of 0.207, whereas *EF1α* performed poorly. In set 3, *SAND* and *PGK* were the most highly ranked with an M value of 0.247, while the least stable gene was *UBQ*. *TIP41* and *PP2A-1* performed best both in set 4 and set 5, whereas *ACT* and *GAPDH* had the largest M value, indicating that these genes were the least stably expressed. In conclusion, *TIP41* and *SAND* showed the highest stability among all samples, and *GAPDH* was the least stable reference gene.

geNorm also calculates the pairwise variation (V) to determine the optimal number of reference genes for reliable normalization. If the Vn/n+1 value is lower than the threshold of 0.15, the minimum number of the most suitable reference genes is n. The V2/3 values for set 1 to set 5 were far lower than 0.15, indicating that one stable reference gene is sufficient to obtain accurate results. The V2/3 value (0.138) of the total set demonstrated that two reference genes were suitable for normalization of all the samples (Fig 4B).

#### NormFinder analysis

The ranking orders of candidate reference genes in all sets determined by stability value calculated using NormFinder are shown in Table 3, with lower value indicating higher stability. The ranking orders determined by this method were similar to the results generated by geNorm. *TIP41* was the most stable genes among all tested samples both in geNorm and NormFinder analysis. For set 1 and set 2, the top two most stable and the least stable reference genes generated by NormFinder were highly consistent with those determined by geNorm. In set 3 and set 4, the three most stable genes and the two least stable gene generated by NormFinder were almost the same as those generated by geNorm. NormFinder determined *PGK* and *EF1α* as the two most stable genes in sample set 5, whereas *PGK* and *EF1α* were ranked sixth and fifth by geNorm, respectively.

**Table 3:** Ranking order of candidate reference genes and stability values calculated by NormFinder.

Ranking order	Set 1		Set 2		Set 3		Set 4		Set 5		Total	
	Gene	Stability	Gene	Stability	Gene	Stability	Gene	Stability	Gene	Stability	Gene	Stability
1	*SAND*	0.050	*PGK*	0.073	*TIP41*	0.073	*PGK*	0.034	*PGK*	0.041	*TIP41*	0.141
2	*GAPDH*	0.056	*SAND*	0.137	*SAND*	0.141	*PP2A-1*	0.088	*EF1α*	0.041	*PP2A-2*	0.185
3	*PP2A-2*	0.057	*PP2A-2*	0.168	*PGK*	0.159	*TIP41*	0.090	*TIP41*	0.051	*PGK*	0.233
4	*PGK*	0.062	*PP2A-1*	0.205	*SKIP16*	0.160	*EF1α*	0.105	*PP2A-1*	0.069	*MTP*	0.292
5	*PP2A-1*	0.086	*TIP41*	0.246	*EF1α*	0.196	*SAND*	0.124	*MTP*	0.079	*SAND*	0.304
6	*ACT*	0.090	*GAPDH*	0.248	*GAPDH*	0.221	*MTP*	0.139	*PP2A-2*	0.094	*EF1α*	0.366
7	*EF1α*	0.092	*MTP*	0.264	*PP2A-2*	0.227	*PP2A-2*	0.147	*UBQ*	0.126	*PP2A-1*	0.399
8	*TIP41*	0.092	*SKIP16*	0.331	*PP2A-1*	0.249	*SKIP16*	0.167	*SAND*	0.132	*SKIP16*	0.463
9	*UBQ*	0.098	*UBQ*	0.346	*ACT*	0.261	*UBQ*	0.211	*SKIP16*	0.176	*UBQ*	0.540
10	*MTP*	0.098	*ACT*	0.354	*MTP*	0.270	*ACT*	0.449	*GAPDH*	0.546	*ACT*	0.669
11	*SKIP16*	0.102	*EF1α*	0.484	*UBQ*	0.325	*GAPDH*	0.480	*ACT*	0.734	*GAPDH*	1.351

#### BestKeeper analysis

BestKeeper ranks the candidate reference genes based on the standard deviation (SD) and the coefficient of variation (CV) of their Cq values [26]. Lower SD and CV values represent higher stability. If the SD values of reference genes are larger than 1.0, these reference genes are considered to be unsuitable for gene expression normalization. Our results showed that almost all the reference genes had SD values smaller than 1.0 except *GAPDH* in all samples (Table 4). The ranking orders were different from the results calculated by GeNorm and NormFinder. For set 1, *SKIP16* was the most stable gene by BestKeeper, while in geNorm and NormFinder analysis, *SKIP16* was the least stable gene. In set 3, *UBQ* ranked second, whereas it ranked at the bottom both in geNorm and NormFinder analysis. For set 2, set 4, set 5 and total, the top three most stable and the least stable reference genes determined by BestKeeper were similar to those generated by geNorm and NormFinder (Fig 4A, Tables 3 and 4).

**Table 4:** Ranking order of candidate reference genes and their SD calculated by BestKeeper.

Ranking order	Set 1		Set 2		Set 3		Set 4		Set 5		Total	
	Gene	CV±SD	Gene	CV±SD	Gene	CV±SD	Gene	CV±SD	Gene	CV±SD	Gene	CV±SD
1	*SKIP16*	0.79±0.20	*SAND*	0.53±0.12	*TIP41*	0.96±0.23	*PP2A-1*	0.50±0.12	*PP2A-1*	0.60±0.14	*TIP41*	1.01±0.23
2	*ACT*	0.75±0.21	*PP2A-1*	0.64±0.15	*UBQ*	1.37±0.28	*SAND*	0.53±0.13	*TIP41*	0.63±0.15	*SAND*	0.97±0.23
3	*PP2A-1*	0.87±0.21	*PGK*	0.69±0.17	*GAPDH*	1.47±0.30	*TIP41*	0.57±0.13	*UBQ*	1.07±0.20	*MTP*	1.58±0.37
4	*PGK*	1.01±0.26	*TIP41*	1.21±0.27	*PP2A-1*	1.20±0.31	*PGK*	0.62±0.16	*SAND*	0.84±0.20	*PP2A-2*	1.56±0.39
5	*MTP*	1.14±0.28	*PP2A-2*	1.28±0.31	*SAND*	1.37±0.35	*MTP*	0.75±0.17	*MTP*	0.96±0.22	*PP2A-1*	1.66±0.40
6	*UBQ*	1.40±0.28	*UBQ*	1.68±0.31	*MTP*	1.49±0.36	*SKIP16*	0.82±0.20	*EF1α*	1.00±0.26	*PGK*	1.84±0.47
7	*SAND*	1.16±0.30	*SKIP16*	1.37±0.33	*EF1α*	1.47±0.39	*PP2A-2*	0.84±0.21	*PGK*	1.11±0.29	*UBQ*	2.52±0.48
8	*PP2A-2*	1.19±0.30	*ACT*	1.21±0.33	*PP2A-2*	1.56±0.39	*EF1α*	0.91±0.24	*SKIP16*	1.16±0.30	*EF1α*	2.23±0.59
9	*GAPDH*	1.54±0.32	*GAPDH*	1.91±0.34	*SKIP16*	1.58±0.39	*UBQ*	1.70±0.33	*PP2A-2*	1.33±0.33	*SKIP16*	3.05±0.76
10	*EF1α*	1.28±0.34	*MTP*	1.52±0.35	*PGK*	1.59±0.42	*ACT*	1.92±0.51	*GAPDH*	2.66±0.62	*ACT*	3.48±0.94
11	*TIP41*	1.56±0.38	*EF1α*	2.56±0.66	*ACT*	1.66±0.45	*GAPDH*	2.46±0.53	*ACT*	3.27±0.90	*GAPDH*	8.43±1.76

#### RefFinder analysis

Finally, RefFinder was used to generate a comprehensive evaluation of candidate reference genes by integrating three distinct algorithms (geNorm, NormFinder and BestKeeper) [40]. The ranking orders determined by RefFinder are depicted in Table 5. Though some differences in ranking orders were found among four programs, the most stable genes were roughly identical. *SAND* was found to be the most stably expressed gene in set 1. *PGK* ranked the first in set 2, and *PP2A-1* ranked the first in set 4. *TIP41* was the most stable gene in set 3, set 5 and total samples. The least stable reference genes in set 1 and set 2 were *UBQ* and *EF1α*, respectively. *ACT* was the least stably expressed gene in set 3 and set 5, as well as *GAPDH* in set 4 and total samples.

**Table 5:** Stability ranking of eleven candidate reference genes based on RefFinder analysis.

Ranking	1	2	3	4	5	6	7	8	9	10	11
**Set 1**											
GeNorm	*GAPDH | SAND*		*EF1α*	*PP2A-2*	*TIP41*	*PGK*	*MTP*	*PP2A-1*	*UBQ*	*ACT*	*SKIP16*
NormFinder	*SAND*	*GAPDH*	*PP2A-2*	*PGK*	*PP2A-1*	*EF1α*	*TIP41*	*ACT*	*MTP*	*UBQ*	*SKIP16*
BestKeeper	*SKIP16*	*ACT*	*PP2A-1*	*PGK*	*UBQ*	*MTP*	*SAND*	*PP2A-2*	*GAPDH*	*EF1α*	*TIP41*
Recommended comprehensive ranking	*SAND*	*GAPDH*	*PP2A-2*	*PGK*	*PP2A-1*	*EF1α*	*ACT*	*SKIP16*	*TIP41*	*MTP*	*UBQ*
**Set 2**											
GeNorm	*PGK | SAND*		*PP2A-1*	*TIP41*	*PP2A-2*	*GAPDH*	*MTP*	*UBQ*	*SKIP16*	*ACT*	*EF1α*
NormFinder	*PGK*	*SAND*	*PP2A-2*	*PP2A-1*	*TIP41*	*GAPDH*	*MTP*	*SKIP16*	*UBQ*	*ACT*	*EF1α*
BestKeeper	*SAND*	*PP2A-1*	*PGK*	*TIP41*	*PP2A-2*	*UBQ*	*SKIP16*	*ACT*	*GAPDH*	*MTP*	*EF1α*
Recommended comprehensive ranking	*PGK*	*SAND*	*PP2A-1*	*PP2A-2*	*TIP41*	*GAPDH*	*MTP*	*UBQ*	*SKIP16*	*ACT*	*EF1α*
**Set 3**											
GeNorm	*PGK | SAND*		*TIP41*	*SKIP16*	*PP2A-2*	*EF1α*	*GAPDH*	*ACT*	*PP2A-1*	*MTP*	*UBQ*
NormFinder	*TIP41*	*SAND*	*PGK*	*SKIP16*	*EF1α*	*GAPDH*	*PP2A-2*	*PP2A-1*	*ACT*	*MTP*	*UBQ*
BestKeeper	*TIP41*	*UBQ*	*GAPDH*	*PP2A-1*	*SAND*	*MTP*	*EF1α*	*PP2A-2*	*SKIP16*	*PGK*	*ACT*
Recommended comprehensive ranking	*TIP41*	*SAND*	*PGK*	*SKIP16*	*GAPDH*	*EF1α*	*PP2A-2*	*PP2A-1*	*UBQ*	*MTP*	*ACT*
**Set 4**											
GeNorm	*PP2A-1 | TIP41*		*PGK*	*PP2A-2*	*SAND*	*EF1α*	*MTP*	*SKIP16*	*UBQ*	*ACT*	*GAPDH*
NormFinder	*PGK*	*PP2A-1*	*TIP41*	*EF1α*	*SAND*	*MTP*	*PP2A-2*	*SKIP16*	*UBQ*	*ACT*	*GAPDH*
BestKeeper	*PP2A-1*	*SAND*	*TIP41*	*PGK*	*MTP*	*SKIP16*	*PP2A-2*	*EF1α*	*UBQ*	*ACT*	*GAPDH*
Recommended comprehensive ranking	*PP2A-1*	*PGK*	*TIP41*	*SAND*	*EF1α*	*MTP*	*PP2A-2*	*SKIP16*	*UBQ*	*ACT*	*GAPDH*
**Set 5**											
GeNorm	*PP2A-1 | TIP41*		*UBQ*	*MTP*	*EF1α*	*PGK*	*SAND*	*PP2A-2*	*SKIP16*	*GAPDH*	*ACT*
NormFinder	*PGK*	*EF1α*	*TIP41*	*PP2A-1*	*MTP*	*PP2A-2*	*UBQ*	*SAND*	*SKIP16*	*GAPDH*	*ACT*
BestKeeper	*PP2A-1*	*TIP41*	*UBQ*	*SAND*	*MTP*	*EF1α*	*PGK*	*SKIP16*	*PP2A-2*	*GAPDH*	*ACT*
Recommended comprehensive ranking	*TIP41*	*PP2A-1*	*EF1α*	*PGK*	*UBQ*	*MTP*	*SAND*	*PP2A-2*	*SKIP16*	*GAPDH*	*ACT*
**Total**											
GeNorm	*TIP41 | SAND*		*PP2A-2*	*MTP*	*PP2A-1*	*UBQ*	*PGK*	*EF1α*	*SKIP16*	*ACT*	*GAPDH*
NormFinder	*TIP41*	*PP2A-2*	*PGK*	*MTP*	*SAND*	*EF1α*	*PP2A-1*	*SKIP16*	*UBQ*	*ACT*	*GAPDH*
BestKeeper	*TIP41*	*SAND*	*MTP*	*PP2A-2*	*PP2A-1*	*PGK*	*UBQ*	*EF1α*	*SKIP16*	*ACT*	*GAPDH*
Recommended comprehensive ranking	*TIP41*	*PP2A-2*	*SAND*	*MTP*	*PGK*	*PP2A-1*	*EF1α*	*UBQ*	*SKIP16*	*ACT*	*GAPDH*

### Validation of selected reference genes by expression analysis of target genes

**Fig 5:**
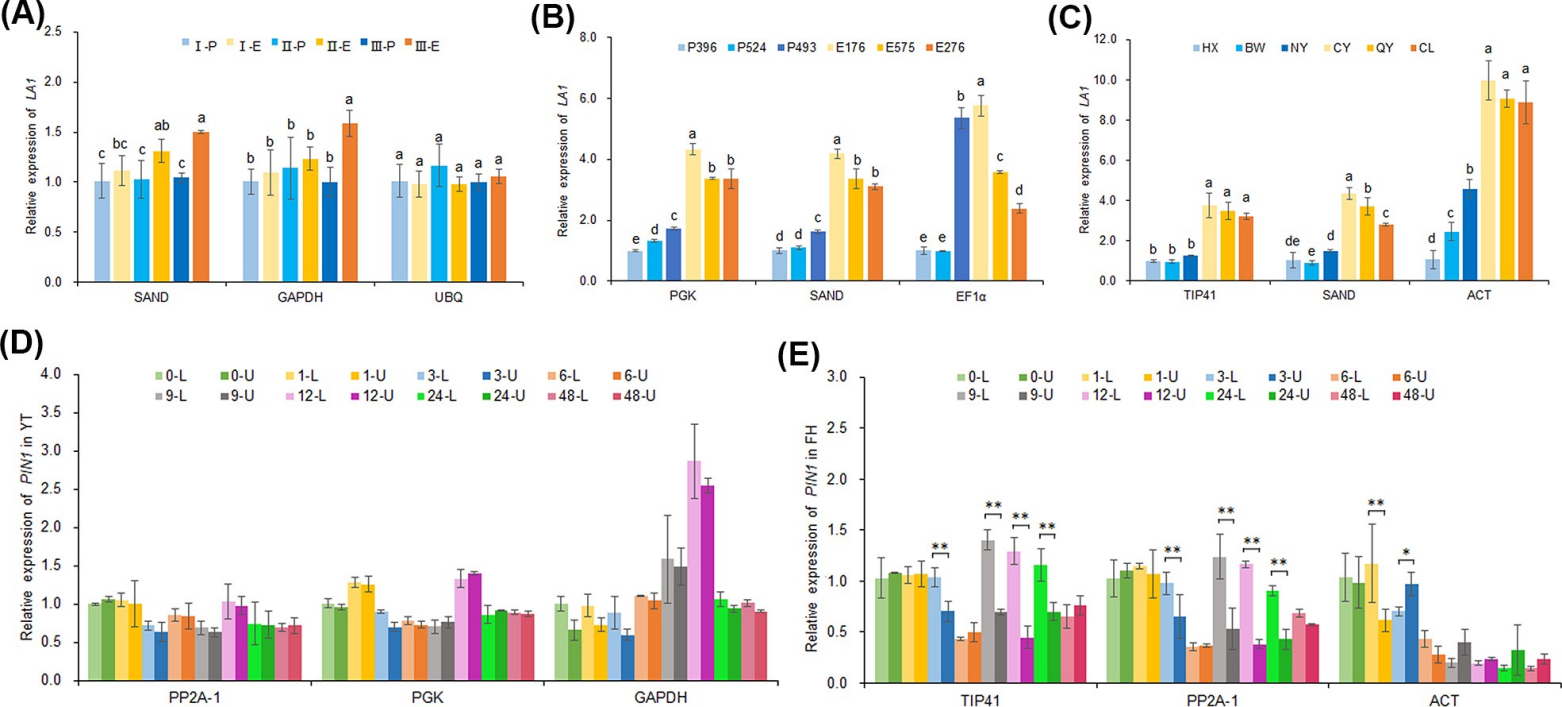
The expression profiles of two target genes normalized by stable and unstable reference genes. (**A**) The expression pattern of *LA1* of prostrate and erect bulk at three different shoot development stages; (**B**) Expression levels of *LA1* in prostrate and erect strains; (**C**) Expression levels of *LA1* in new cultivars of ground-cover chrysanthemum; (**D**) The expression pattern of *PIN1* during the process of gravitropic response in YT; (**E**) The expression pattern of *PIN1* during the process of gravitropic response in FH. I, Stage I; II, Stage II; III, Stage III; P, prostrate; E, erect; HX, ‘Fukanhongxiu’, CNA20170986; BW, ‘Fukanbowu’, CNA20170990; NY, ‘Fukannongyun’, CNA20170985; CY, ‘Fukanchiyan’, CNA20170989; QY, ‘Beilinqiuyun’, CNA20170987; CL, ‘Fukanchenlu’, CNA20170988; L, the lower half of stem; U, the upper half of stem. Error bars represent SD, n = 3. *, P < 0.05, **, P < 0.01, Student t test.

Previous studies have shown that *LAZY1* (*LA1*) is the principal determinant of branch angle that mediate plant architecture [18,29,30,31–34]. *PIN1*, one of the *PIN-FORMED* (*PIN*) family members, encodes an auxin efflux carrier involved in the auxin redistribution in gravitropic response [35,36]. Melting curve and amplification efficiencies of these target genes are shown in S3 Fig. To verify the stability of the screened reference genes, the expression patterns of these target genes were analyzed using two most stable and one least stable reference genes based on the results of four programs. In set 1, when normalized using the most stable reference genes *SAND*, the relative expression level of *CmLA1* increased in the erect bulk during the process of shoot development, while it basically remained unchanged in prostrate bulk among three developmental stages. The expression pattern of *CmLA1* normalized with the second most stable reference gene *GAPDH* displayed similar trends with that normalized with *SAND*. However, when normalized by the least stable gene *UBQ*, the expression profile showed significant differences (Fig 5A). In set 2 and set 3, when normalized with the two most stable reference genes, the expression patters of *CmLA1* were almost identical, with the transcript level three times higher in vertical strains and cultivars as compared to prostrate plants. Whereas, the expression pattern exhibited obvious discrepancies when normalized by the least stable gene (Fig 5B and 5C). During the process of gravitropic response in YT, when normalized with two most stable reference genes *PP2A-1* and *PGK*, similar expression patterns were generated that relative expression levels of *PIN1* in the upper and lower half of stem were almost the same. While, the transcription profile of *PIN1* was contradictory to the above patterns when normalized using *GAPDH*, the least stable gene (Fig 5D). In FH (a vertical cultivar of ground-cover chrysanthemum ‘Fanhuasijin’, CNA20090874), the expression pattern of *PIN1* normalized with *TIP41*, when subjected to gravistimulation, was almost the same as that normalized by *PP2A-1*. The transcript abundance of *PIN1* in the lower half of stem was significantly higher than that in the upper half at 3, 9, 12, and 24 h after horizontal treatment. Nevertheless, no obvious differences were observed between the upper and lower half of stem when normalized by the least stable gene *ACT* (Fig 5E). Taken together, the significant discrepancies in expression level of *PIN1* might be responsible for the differences in gravitropic response between YT and FH. These results verified the reliability of screened reference genes and revealed that the accuracy of qRT-PCR analysis could be altered when using the least stable reference gene.

## Discussion

### The significance of systematic evaluation of reference genes in shoot development and gravitropic response in chrysanthemum

The qRT-PCR has become a forceful method to analyze gene expression, however its accuracy mainly relys on suitable reference genes [1–3]. Previous studies had demonstrated that no universal reference genes could express steadily in various organisms and circumstances and that contradictory results could be generated while using unsuitable reference genes [4,5,7]. Several genes had been evaluated as suitable reference genes for chrysanthemum in previous studies focused on biotic and abiotic stress [8], photoperiodic treatments [38], cross-ploidy level comparisons [6], flower color [39], and floral development [9]. However, no detailed verification has been conducted on whether these reference genes were suitable for data normalizing during shoot development and gravitropic response. The traditional housekeeping gene *ACT*, which has been considered as a reliable reference gene for many years, was used for data normalization in real-time PCR during shoot graviresponse in chrysanthemum cultivar ‘Yuhuajinhua’ [23]. Whereas, in this research, *ACT* had poor performance in most of the sample sets, which was consistent with studies in chrysanthemum cultivar ‘Zhongshanzigui’ [8], *Chrysanthemum lavandulifolium* [38], and *Lagerstroemia speciose* [41]. Therefore, a systematic evaluation of reference genes is extremely important before analyzing the expression patterns of key genes involved in shoot development and gravitropic response in prostrate and erect chrysanthemum.

### The high efficiency of candidate reference gene selection based on transcriptome data

The commonly-used approaches for selecting candidate reference genes are literature reviewing and public database searching for housekeeping genes related to certain biological processes such as glycolysis, cellular metabolism, protein synthesis and degradation [42]. Several studies have shown that the expression of these reference genes might vary to a great extent under different experimental conditions [43]. Thus, unreliable results may be generated when blindly selecting these reference genes as candidates without any other expression data [7,24]. Expression data of thousands of genes can be obtained from transcriptome sequencing, which is an important method for gene expression studies because of its high throughput, accuracy and efficiency [44,45]. Therefore, through analyzing the transcriptome data, the reference genes stably expressed in different cultivars, tissues, and under various experimental conditions can be screened out. This method has been successfully applied to several studies in plant species such as *Prunus mume* [2], seashore paspalum [40], and chrysanthemum [6,38,39]. In the present study, based on transcriptome data, eleven candidate reference genes with relatively stable expression (|log2Ratio| < 0.3) were screened out. Another four reference genes, including *PSAA*, *F-box*, *TUA* (*α-tublin*), and *TUB* (*β-tublin*), were eliminated because of their wide variation in expression. This effective approach to identify candidate reference genes based on transcriptome libraries provided a solid foundation for further evaluating the expression stability of these candidates.

### The superiority of a comprehensive evaluation of stability of reference genes using four programs

Previous studies have revealed that there is no consensus on which kind of statistical program should be used to analyze the stability of reference genes because different programs based on distinct algorithms generate potentially conflicting results [9]. In the research of tobacco, two programs (geNorm and NormFinder) were used to evaluate the stability of reference genes, however, the ranking orders were inconsistent in stress-treated sample set [46]. This suggests that it is necessary to use at least three different algorithms to obtain reliable results. Multi-algorithm analysis had been performed to select suitable reference genes under different experimental situations in plants such as *Chrysanthemum* [9], *Rhododendron molle* [47], tree peony [48], seashore paspalum [40], and *Lagerstroemia* [41]. Therefore, in the present study, four computational programs (geNorm, NormFinder, BestKeeper, and RefFinder) were applied to evaluating stability of candidate reference genes. The results of geNorm and NormFinder were similar, but they exhibited quite differences from those of BestKeeper. In set 1, geNorm and NormFinder determined *SAND* and *GAPDH* as most stable genes and *SKIP16* as the least stable gene, while *SKIP16* was the most stable gene in BestKeeper analysis. But when we used RefFinder to integrate the ranking orders generated by these three algorithms, *SAND* and *GAPDH* were still the most stable reference genes. These results coincided with studies on flower development in *Chrysanthemum* [9] and *Lagerstroemia* [41]. Hence, a comprehensive analysis using four programs can generate more reliable reference genes.

### The stability of candidate reference genes used in shoot development and gravitropic response in chrysanthemum

By interpreting results from four frequently-used methods (geNorm, NormFinder, BestKeeper, and RefFinder), several stable reference genes were identified in this study, including *SAND*, *PP2A-1*, and *TIP41*. The SAND family proteins play major roles in the downstream regulatory pathway of endocytic transport [49]. The current study determined *SAND* as the best candidate for normalization in different chrysanthemum genotypes during different shoot developmental stages. *SAND* have also been reported as an optimal reference gene in previous researches, such as in *Petunia hybrida* during leaf and flower development [50], in different citrus organs and following different biotic stresses [51], in vegetative tissues and organs during berry development [52], in *Chrysanthemum* during flower development [9], and in salt-treated seashore paspalum [40]. *PP2A-1*, which encodes the serine/threonine phosphatase, plays a prominent role in metabolism, DNA replication, transcription and translation, cell cycle, and signal transduction [53]. It was used as a superior reference gene in different cotton plant organs [54], different color lines during flower developmental stages of cineraria [55], and cold-treated seashore paspalum [40]. *TIP41* (*TAP42 INTERACTING PROTEIN OF 41 kDa*), which modifies cell growth in response to nutrient status and environmental conditions [56], has been revealed as an appropriate reference gene in whole tomato developmental process [57]. In our study, *PP2A-1* and *TIP41* were stably expressed in set 4 and set 5, thus determining them as superior reference genes in gravitropic response of YT and FH. The traditional reference gene *ACT* was used for data normalization in qPCR analysis during graviresponse in Arabidopsis seedlings [58], peanut gynophores [20] and chrysanthemum shoot [23]. However, in the present study, the expression of *ACT* displayed unacceptable variation during shoot graviresponse. The variable expression of *ACT* may closely related to the involvement of cytoskeleton in the gravitropic bending growth [23]. *ACT* may regulate the asymmetric elongation of the upper and lower half of the bending part of the stem, so it inappropriate to use *ACT* as reference gene during shoot gravity response.

### The reliability of comprehensive evaluation on reference genes

Expression patterns of target genes were found to vary significantly when normalized by stable and unstable reference genes, which led to conflicting results [9]. In this study, to further verify the reliability of the selected reference genes, the expression levels of target genes (*LA1* and *PIN1*) were analyzed using two most stable and one least stable reference genes based on integrating results of four programs. In set 1, set 2, and set 3, when normalizing with the two most stable genes, the expression patterns of *LA1* were similar, which was highly consistent with the results that the relative expression level of *ZmLA1* in *la1-ref* mutant plants was obviously lower than that in wild maize [31]. However, the results were quite different when the least stable gene was used for normalization. During the process of stem graviresponse in YT or FH, transcription levels of *PIN1* were almost the same when normalized with two most stable reference genes, while the expression pattern was contradictory to the above outcome when normalized using the least stable gene. The significant discrepancies in expression level of *PIN1* might be responsible for the differences in gravitropic response between YT and FH. The weaker gravitropic response in YT might be due to the reduced asymmetric distribution of IAA which is caused by the absence of significant discrepancies in expression level of *PIN1* between the upper and lower half of stem. These results validated the reliability of screened reference genes and also indicated the importance of selecting suitable internal control genes for qRT-PCR analysis.

## Conclusions

This research provides the first systematic evaluation of reference genes for qRT-PCR analysis in shoot development and gravitropic response of prostrate and erect chrysanthemum. In general, *TIP41* was the most stable gene for all the samples. *SAND* could be applied as a superior reference gene in different genotypes and during the process of stem development. The suitable reference genes for gravitropic response could be *PP2A-1*. The expression patterns of *LA1* and *PIN1* further verified the importance of selection of suitable reference genes for qRT-PCR analysis. Specific conclusions drawn from this study could provide more accurate and reliable qRT-PCR normalization for future studies on the expression patterns of genes regulating plant architecture of chrysanthemums.

## Supporting information

S1 Fig Melting curve and melting peak of candidate reference genes. (TIF)

S2 Fig Standard curve of candidate reference genes in the five-fold serial dilution. (TIF)

S3 Fig Melting curve and standard curve of target genes. (TIF)

S1 Table FPKM and log2Ratio (III_P vs III_E) of candidate reference genes. (DOCX)

S2 Table Primer sequences for gene cloning of reference genes. (DOCX)

## References

[1] Udvardi MK, Czechowski T, Scheible WR (2008) Eleven golden rules of quantitative RT-PCR. Plant Cell 20: 1736–1737. 10.1105/tpc.108.061143 18664613 PMC2518243

[2] Wang T, Lu J, Xu Z, Yang W, Wang J, Cheng T, et al (2014) Selection of suitable reference genes for miRNA expression normalization by qRT-PCR during flower development and different genotypes of *Prunus mume*. Sci Hortic 169: 130–137.

[3] Czechowski T, Stitt M, Altmann T, Udvardi MK, Scheible WR (2005) Genome-wide identification and testing of superior reference genes for transcript normalization in Arabidopsis. Plant Physiol 139: 5–17. 10.1104/pp.105.063743 16166256 PMC1203353

[4] Hu Y, Fu H, Qiao H, Sun S, Zhang W, Jin S, et al (2018) Validation and evaluation of reference genes for quantitative real-time PCR in *Macrobrachium Nipponense*. Int J Mol Sci 19: 2258. 10.3390/ijms19082258 PMC6121487 30071669

[5] Dheda K, Huggett JF, Chang J, Kim LU, Bustin SA, Johnson MA, et al (2005) The implications of using an inappropriate reference gene for real-time reverse transcription PCR data normalization. Anal Biochem 344: 141–143. 10.1016/j.ab.2005.05.022 16054107

[6] Wang H, Chen S, Jiang J, Zhang F, Chen F (2015) Reference gene selection for cross-species and cross-ploidy level comparisons in *Chrysanthemum* spp. Sci Rep 5: 8094 10.1038/srep08094 25627791 PMC4308696

[7] Huggett J, Dheda K, Bustin S, Zumla A (2005) Real-time RT-PCR normalisation; strategies and considerations. Genes Immun 6: 279–284. 10.1038/sj.gene.6364190 15815687

[8] Gu C, Chen S, Liu Z, Shan H, Luo H, Guan Z, et al (2011) Reference gene selection for quantitative real-time PCR in Chrysanthemum subjected to biotic and abiotic stress. Mol Biotechnol 49: 192–197. 10.1007/s12033-011-9394-6 21416201

[9] Qi S, Yang L, Wen X, Hong Y, Song X, Zhang M, et al (2016) Reference gene selection for RT-qPCR analysis of flower development in *Chrysanthemum morifolium* and *Chrysanthemum lavandulifolium*. Front Plant Sci 7: 287 10.3389/fpls.2016.00287 27014310 PMC4786574

[10] Anderson NO (2007) Flower breeding and genetics—issues, challenges and opportunities for the 21st century 2nd ed. Springer-Verlag, New York, United States; 389–437.

[11] Dai S, Wang W, Li M, Xu Y (2005) Phylogenetic relationship of *Dendranthema* (DC.) Des Moul. revealed by fluorescent in situ hybridization. J Integr Plant Biol 47: 783–791.

[12] Huang D, Li X, Sun M, Zhang T, Pan H, Cheng T, et al (2016) Identification and characterization of CYC-Like genes in regulation of ray floret development in *Chrysanthemum morifolium*. Front Plant Sci 7: 1633 10.3389/fpls.2016.01633 27872631 PMC5097909

[13] Liu H, Sun M, Du D, Pan H, Cheng T, Wang J, et al (2016) Whole-transcriptome analysis of differentially expressed genes in the ray florets and disc florets of *Chrysanthemum morifolium*. BMC Genomics 17: 398 10.1186/s12864-016-2733-z 27225275 PMC4881213

[14] Wang P, Chen J (1990) Studies on breeding ground-cover chrysanthemum new cultivars. Acta Hortic Sin 8: 223–228.

[15] Chen F, Fang W, Zhao H, Guan Z, Xu G (2005) New varieties of chrysanthemum-ground-cover varieties. Acta Hortic Sin 32: 1167.

[16] Chen J, Zhong J, Shi X, Zhang Q, Sun M (2018) *Chrysanthemum yantaiense*, a rare new species of Asteraceae from China. Phytotaxa 374: 92.

[17] Withers JC, Shipp MJ, Rupasinghe SG, Sukumar P, Schuler MA, Muday GK, et al (2013) *Gravity Persistent Signal 1* (*GPS1*) reveals novel cytochrome P450s involved in gravitropism. Am J Bot 100: 183–193. 10.3732/ajb.1200436 23284057

[18] Zhang N, Yu H, Yu H, Cai Y, Huang L, Xu C, et al (2018) A core regulatory pathway controlling rice tiller angle mediated by the LAZY1-dependent asymmetric distribution of auxin. Plant Cell 30: 1461–1475. 10.1105/tpc.18.00063 29915152 PMC6096585

[19] Hu L, Mei Z, Zang A, Chen H, Dou X, Jin J, et al (2013) Microarray analyses and comparisons of upper or lower flanks of rice shoot base preceding gravitropic bending. PLoS ONE 8: e74646 10.1371/journal.pone.0074646 24040303 PMC3764065

[20] Li H, Chen X, Zhu F, Liu H, Hong Y, Liang X. (2013) Transcriptome profiling of peanut (*Arachis hypogaea*) gynophores in gravitropic response. Funct Plant Biol 40: 1249. 10.1071/FP13075 32481192

[21] Zhang S, Chen S, Chen F, Teng N, Fang W, Guan Z. (2008) Anatomical structure and gravitropic response of the creeping shoots of ground-cover chrysanthemum ‘Yuhuajinhua’. Plant Growth Regul 56: 141–150.

[22] Zhang S, Chen S, Chen F, Liu Z, Fang W. (2012) The regulatory role of the auxin in the creeping chrysanthemum habit. Russ J Plant Physiol 59: 364–371.

[23] Xia S, Chen Y, Jiang J, Chen S, Guan Z, Fang W, et al (2013) Expression profile analysis of genes involved in horizontal gravitropism bending growth in the creeping shoots of ground-cover chrysanthemum by suppression subtractive hybridization. Mol Biol Rep 40: 237–246. 10.1007/s11033-012-2054-5 23065216

[24] Vandesompele J, de Preter K, Pattyn F, Poppe B, van Roy N, de Paepe A, et al (2002) Accurate normalization of real-time quantitative RT-PCR data by geometric averaging of multiple internal control genes. Genome Biol 3: RESEARCH0034. 12184808 10.1186/gb-2002-3-7-research0034 PMC126239

[25] Andersen CL, Jensen JL, Orntoft TF (2004) Normalization of real-time quantitative reverse transcription-PCR data: a model-based variance estimation approach to identify genes suited for normalization, applied to bladder and colon cancer data sets. Cancer Res 64: 5245–5250. 10.1158/0008-5472.CAN-04-0496 15289330

[26] Pfaffl MW, Tichopad A, Prgomet C, Neuvians TP (2004) Determination of stable housekeeping genes, differentially regulated target genes and sample integrity: BestKeeper-Excel-based tool using pair-wise correlations. Biotechnol Lett 26: 509–515. 10.1023/b:bile.0000019559.84305.47 15127793

[27] Livak KJ, Schmittgen TD (2001) Analysis of relative gene expression data using real-time quantitative PCR and the 2^-ΔΔ^CT method. Methods 25: 402–408. 10.1006/meth.2001.1262 11846609

[28] Hellemans J, Mortier G, De Paepe A, Speleman F, Vandesompele J (2007) qBase relative quantification framework and software for management and automated analysis of real-time quantitative PCR data. Genome Biol 8: R19 10.1186/gb-2007-8-2-r19 17291332 PMC1852402

[29] Li P, Wang Y, Qian Q, Fu Z, Wang M, Zeng D, et al (2007) LAZY1 controls rice shoot gravitropism through regulating polar auxin transport. Cell Res 17: 402–410. 10.1038/cr.2007.38 17468779

[30] Yoshihara T, Iino M (2007) Identification of the gravitropism-related rice gene LAZY1 and elucidation of LAZY1-dependent and -independent gravity signaling pathways. Plant Cell Physiol 48: 678–688. 10.1093/pcp/pcm042 17412736

[31] Dong Z, Jiang C, Chen X, Zhang T, Ding L, Song W, et al (2013) Maize LAZY1 mediates shoot gravitropism and inflorescence development through regulating auxin transport, auxin signaling, and light response. Plant Physiol 163: 1306–1322. 10.1104/pp.113.227314 24089437 PMC3813652

[32] Yoshihara T, Spalding EP, Iino M (2013) AtLAZY1 is a signaling component required for gravitropism of the Arabidopsis thaliana inflorescence. Plant J 74: 267–279. 10.1111/tpj.12118 23331961

[33] Taniguchi M, Furutani M, Nishimura T, Nakamura M, Fushita T, Iijima K, et al (2017) The Arabidopsis LAZY1 family plays a key role in gravity signaling within statocytes and in branch angle control of roots and shoots. Plant Cell 29: 1984–1999. 10.1105/tpc.16.00575 28765510 PMC5590491

[34] Yoshihara T, Spalding EP (2017) LAZY genes mediate the effects of gravity on auxin gradients and plant architecture. Plant Physiol 175: 959–969. 10.1104/pp.17.00942 28821594 PMC5619908

[35] Noh B, Bandyopadhyay A, Peer WA, Spalding EP, Murphy AS (2003) Enhanced gravi- and phototropism in plant *mdr* mutants mislocalizing the auxin efflux protein PIN1. Nature, 423: 999–1002. 10.1038/nature01716 12827205

[36] Wisniewska J, Xu J, Seifertova D, Brewer PB, Ruzicka K, Blilou I, et al (2006) Polar PIN localization directs auxin flow in plants. Science 312: 883 10.1126/science.1121356 16601151

[37] Remans T, Smeets K, Opdenakker K, Mathijsen D, Vangronsveld J, Cuypers A. (2008) Normalisation of real-time RT-PCR gene expression measurements in *Arabidopsis thaliana* exposed to increased metal concentrations. Planta 227: 1343–1349. 10.1007/s00425-008-0706-4 18273637

[38] Fu J, Wang Y, Huang H, Zhang C, Dai S (2012) Reference gene selection for RT-qPCR analysis of *Chrysanthemum lavandulifolium* during its flowering stages. Mol Breeding 31: 205–215.

[39] Hong Y, Dai S (2015) Selection of reference genes for real-time quantitative polymerase chain reaction analysis of light-dependent anthocyanin biosynthesis in chrysanthemum. J Am Soc Hortic Sci 140: 68–77.

[40] Liu Y, Liu J, Xu L, Lai H, Chen Y, Yang Z, et al (2017) Identification and validation of reference genes for seashore paspalum response to abiotic stresses. Int J Mol Sci 18: 1322. 10.3390/ijms18061322 PMC5486143 28635628

[41] Zheng T, Chen Z, Ju Y, Zhang H, Cai M, Pan H, et al (2018) Reference gene selection for qRT-PCR analysis of flower development in *Lagerstroemia indica* and *L*. *speciosa*. PLoS One 13: e0195004 10.1371/journal.pone.0195004 29579116 PMC5868847

[42] Yeap WC, Loo JM, Wong YC, Kulaveerasingam H (2013) Evaluation of suitable reference genes for qRT-PCR gene expression normalization in reproductive, vegetative tissues and during fruit development in oil palm. Plant Cell Tiss Organ Cult 116: 55–66.

[43] Gutierrez L, Mauriat M, Guenin S, Pelloux J, Lefebvre JF, Louvet R, et al (2008) The lack of a systematic validation of reference genes: a serious pitfall undervalued in reverse transcription-polymerase chain reaction (RT-PCR) analysis in plants. Plant Biotechnol J 6: 609–618. 10.1111/j.1467-7652.2008.00346.x 18433420

[44] Lee JM, Roche JR, Donaghy DJ, Thrush A, Sathish P (2010) Validation of reference genes for quantitative RT-PCR studies of gene expression in perennial ryegrass (*Lolium perenne* L.). BMC Mol Biol 11: 8 10.1186/1471-2199-11-8 20089196 PMC2827471

[45] Martin JA, Wang Z (2011) Next-generation transcriptome assembly. Nat Rev Genet 12: 671–682. 10.1038/nrg3068 21897427

[46] Schmidt GW, Delaney SK (2010) Stable internal reference genes for normalization of real-time RT-PCR in tobacco (Nicotiana tabacum) during development and abiotic stress. Mol Genet Genomics 283: 233–241. 10.1007/s00438-010-0511-1 20098998

[47] Xiao Z, Sun X, Liu X, Li C, He L, Chen S, et al (2016) Selection of reliable reference genes for gene expression studies on Rhododendron molle G. Don. Front Plant Sci 7: 1547 10.3389/fpls.2016.01547 27803707 PMC5067439

[48] Li J, Han J, Hu Y, Yang J (2016) Selection of reference genes for quantitative real-time PCR during flower development in tree peony (*Paeonia suffruticosa* Andr.). Front Plant Sci 7: 516 10.3389/fpls.2016.00516 27148337 PMC4838814

[49] Poteryaev D, Spang A (2005) A role of SAND-family proteins in endocytosis. Biochem Soc Trans 33: 606–608. 10.1042/BST0330606 16042554

[50] Mallona I, Lischewski S, Weiss J, Hause B, Egea-Cortines M (2010) Validation of reference genes for quantitative real-time PCR during leaf and flower development in *Petunia hybrida*. BMC Plant Biol 10: 4 10.1186/1471-2229-10-4 20056000 PMC2827423

[51] Mafra V, Kubo KS, Alves-Ferreira M, Ribeiro-Alves M, Stuart RM, Boava LP, et al (2012) Reference genes for accurate transcript normalization in citrus genotypes under different experimental conditions. PLoS One 7: e31263 10.1371/journal.pone.0031263 22347455 PMC3276578

[52] Ye X, Zhang F, Tao Y, Song S, Fang J (2015) Reference gene selection for quantitative real-time PCR normalization in different cherry genotypes, developmental stages and organs. Sci Hortic 181: 182–188.

[53] Janssens V, Goris J (2001) Protein phosphatase 2A: A highly regulated family of serine/threonine phosphatases implicated in cell growth and signaling. Biochem J 353: 417–439. 10.1042/0264-6021:3530417 11171037 PMC1221586

[54] Artico S, Nardeli SM, Brilhante O, Grossi-de-Sa MF, Alves-Ferreira M (2010) Identification and evaluation of new reference genes in *Gossypium hirsutum* for accurate normalization of real-time quantitative RT-PCR data. BMC Plant Biol 10: 49 10.1186/1471-2229-10-49 20302670 PMC2923523

[55] Jin X, Fu J, Dai S, Sun Y, Hong Y (2013) Reference gene selection for qPCR analysis in cineraria developing flowers. Sci Hort 153: 64–70.

[56] Punzo P, Ruggiero A, Possenti M, Nurcato R, Costa A, Morelli G, et al (2018) The PP2A-interactor TIP41 modulates ABA responses in *Arabidopsis thaliana*. Plant J 94: 991–1009. 10.1111/tpj.13913 29602224

[57] Exposito-Rodriguez M, Borges AA, Borges-Perez A, Perez JA (2008) Selection of internal control genes for quantitative real-time RT-PCR studies during tomato development process. BMC Plant Biol 8: 131 10.1186/1471-2229-8-131 19102748 PMC2629474

[58] Xue S, Zou J, Liu Y, Wang M, Zhang C, Le J (2019) Involvement of BIG5 and BIG3 in BRI1 Trafficking Reveals Diverse Functions of BIG-subfamily ARF-GEFs in Plant Growth and Gravitropism. Int J Mol Sci 20: 2339. 10.3390/ijms20092339 PMC6539719 31083521

