# Supplementary figures and images for: Selection of optimal reference genes for qRT-PCR analysis of shoot development and graviresponse in prostrate and erect chrysanthemums
Source: PLoS One. 2019 Nov 27;14(11):e0225241. doi: 10.1371/journal.pone.0225241 (PMC6880974; doi:10.1371/journal.pone.0225241)

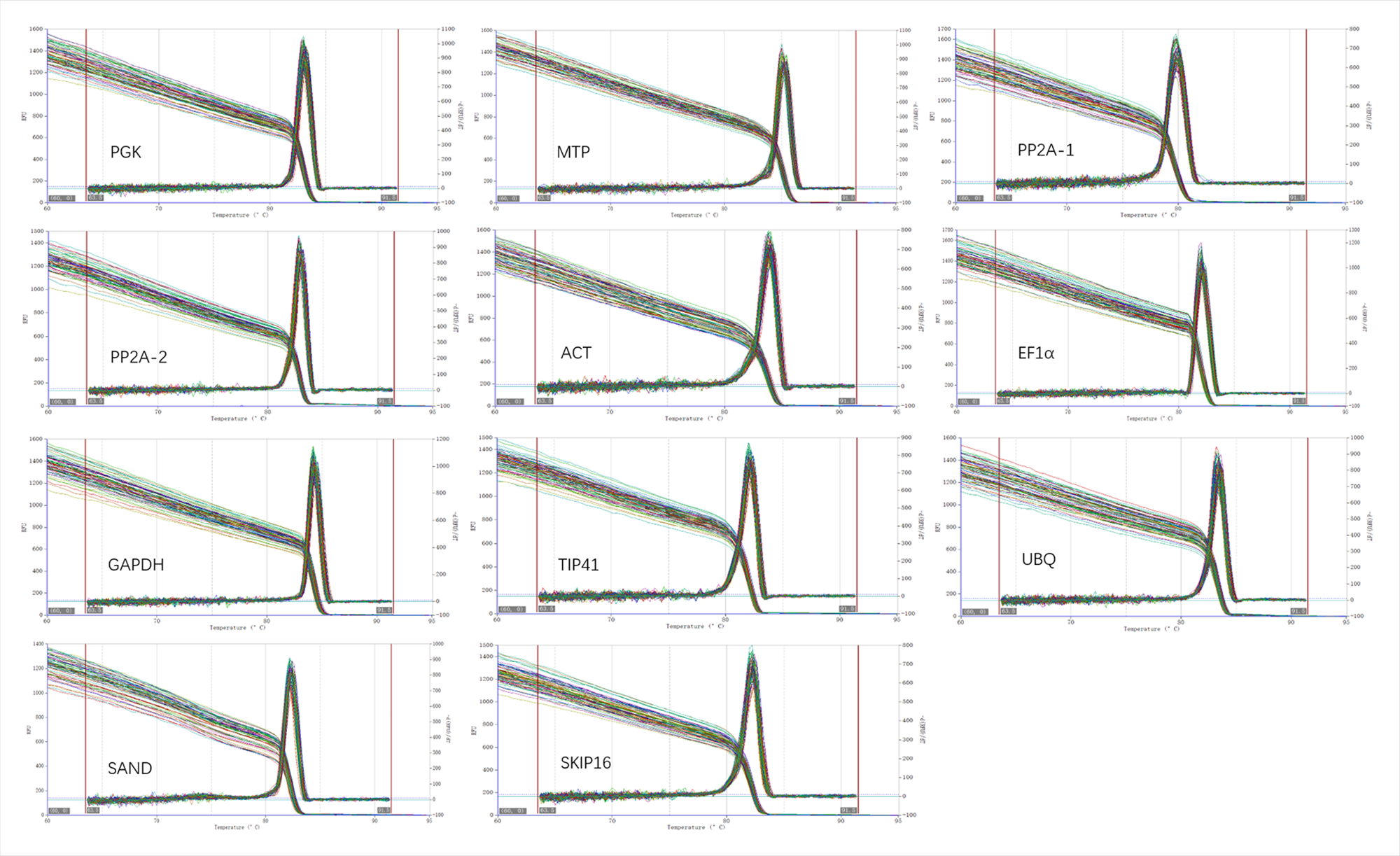

Supplement: S1 Fig — (TIF) [file pone.0225241.s001.tif]

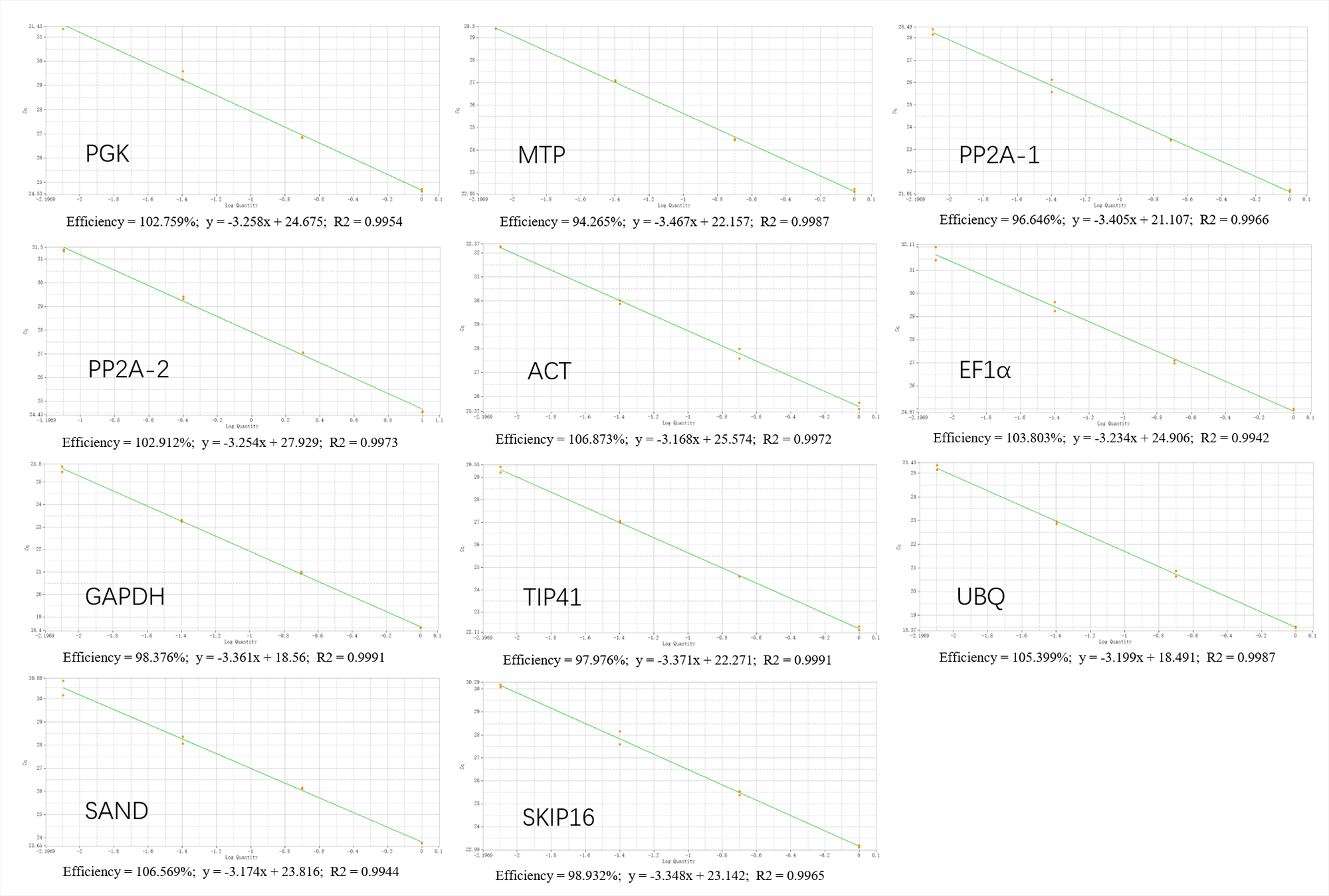

Supplement: S2 Fig — (TIF) [file pone.0225241.s002.tif]

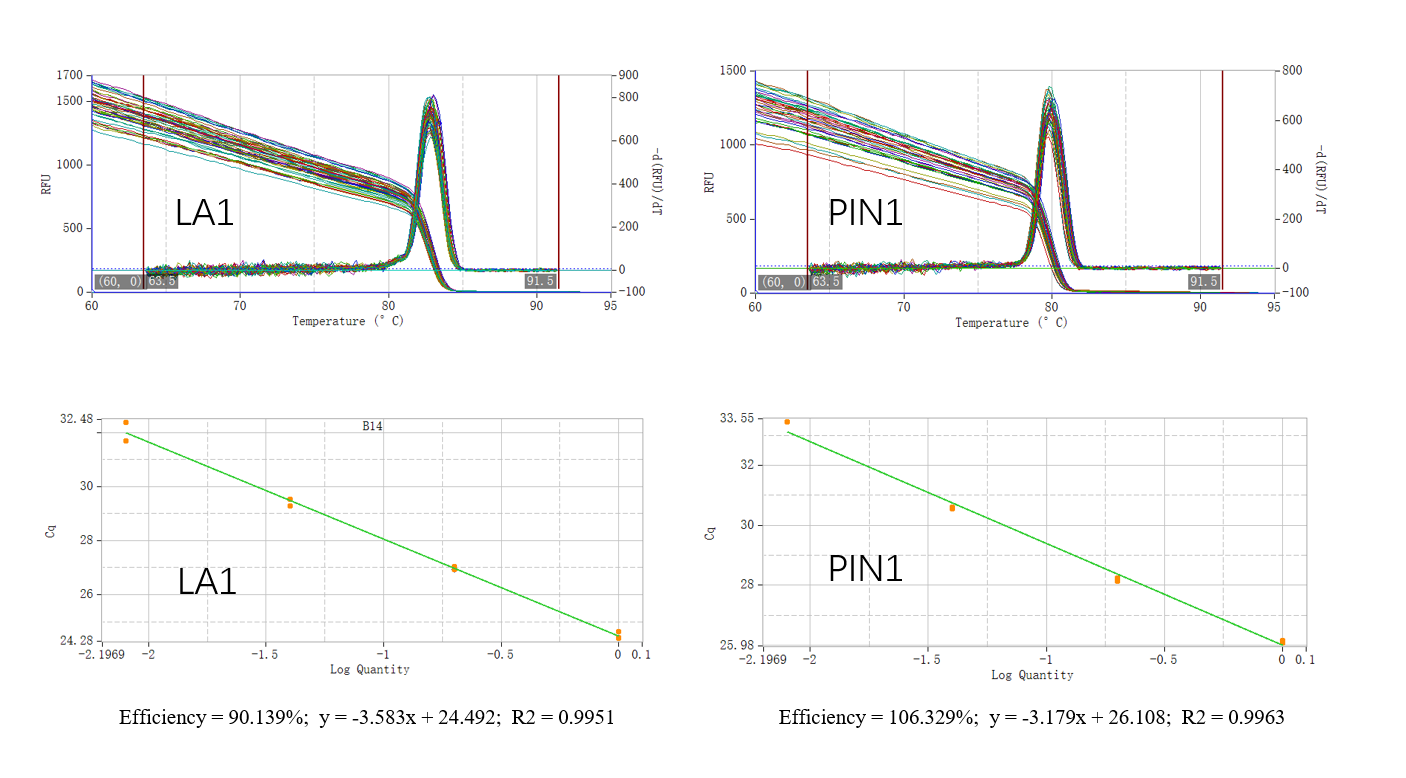

Supplement: S3 Fig — (TIF) [file pone.0225241.s003.tif]
