# Supplementary material for: Selection of optimal reference genes for qRT-PCR analysis of shoot development and graviresponse in prostrate and erect chrysanthemums
Source: PLoS One. 2019 Nov 27;14(11):e0225241. doi: 10.1371/journal.pone.0225241 (PMC6880974; doi:10.1371/journal.pone.0225241)
Supplement: S1 Table — (DOCX) [file pone.0225241.s004.docx]

**S1 Table. FPKM and log2Ratio (III_P vs III_E) of candidate reference genes.**

| **Gene symbol** | **FPKM** | | | | | | \|**log2Ratio**\|  **(III_P vs III_E)** |
| --- | --- | --- | --- | --- | --- | --- | --- |
|  | **III_P_1** | **III_P_2** | **III_P_3** | **III_E_1** | **III_E_2** | **III_E_3** |  |
| *PGK* | 15.3 | 14.7 | 15.1 | 14.6 | 13.7 | 14.8 | 0.089 |
| *MTP* | 57.9 | 59.0 | 58.0 | 60.8 | 59.2 | 56.3 | 0.011 |
| *PP2A-1* | 17.7 | 17.8 | 16.4 | 19.4 | 19.4 | 17.7 | 0.113 |
| *PP2A-2* | 60.0 | 58.8 | 66.5 | 63.2 | 57.2 | 63.8 | 0.022 |
| *ACT* | 320.7 | 349.1 | 395.2 | 388.9 | 352.0 | 360.0 | 0.036 |
| *EF1α* | 13.0 | 12.2 | 14.8 | 11.9 | 14.8 | 14.0 | 0.015 |
| *GAPDH* | 790.9 | 544.0 | 506.4 | 495.4 | 514.7 | 506.9 | 0.298 |
| *TIP41* | 55.6 | 54.2 | 62.9 | 57.8 | 60.4 | 56.1 | 0.002 |
| *UBQ* | 898.7 | 784.7 | 916.5 | 791.3 | 923.3 | 857.1 | 0.039 |
| *SAND* | 22.2 | 25.2 | 23.8 | 22.0 | 23.5 | 22.5 | 0.023 |
| *SKIP16* | 44.6 | 46.7 | 47.2 | 37.2 | 38.5 | 40.0 | 0.272 |
