# Supplementary material for: Selection of optimal reference genes for qRT-PCR analysis of shoot development and graviresponse in prostrate and erect chrysanthemums
Source: PLoS One. 2019 Nov 27;14(11):e0225241. doi: 10.1371/journal.pone.0225241 (PMC6880974; doi:10.1371/journal.pone.0225241)
Supplement: S2 Table — (DOCX) [file pone.0225241.s005.docx]

| **S2 Table. Primer sequences for gene cloning of reference genes.** | | | | |
| --- | --- | --- | --- | --- |
| **Gene symbol** | **Primer sequence (5′–3′) for gene cloning**  **Forward/Reverse** | **Amplicon length (bp)** | **Annealing TM (℃)** |  |
| *PGK* | ACAAGATGATGACCAGATTTC | 806 | 55 |  |
|  | AATCTGTGACTTGAACGAGC |  |  |  |
| *MTP* | ATGGATGATCAGAGGCCGGAAC | 615 | 57 |  |
|  | TCCGTGACCATGATTATCTCCATG |  |  |  |
| *PP2A-1* | ATGATCAAGCAAATACTTGGTAAGG | 1515 | 52 |  |
|  | ATTCACAAGAACAGCTTCATTACT |  |  |  |
| *PP2A-2* | AGAGATGAGCTTGGGTCCAG | 943 | 55 |  |
|  | TAAGAAGTAATCTGGAGTCCTACG |  |  |  |
| *ACTIN* | TGGTCGGAATGGGCCAAAAA | 591 | 57 |  |
|  | AGCAATACCAGGGAACATAGTCG |  |  |  |
| *EF1α* | AAAATGCCGCGCAAAATAAG | 2208 | 53 |  |
|  | CTAGTTCTGCTCGCTTGGTAT |  |  |  |
| *GAPDH* | CCCCAAAGCAAAGAAACATACC | 1239 | 53 |  |
|  | TCACTTCCAGTTATTAGCAACAAT |  |  |  |
| *TIP41* | ATGGAGTGGGAAGCAGACGA | 848 | 57 |  |
|  | TCAAGGGACTTTGAGCTTTTGAG |  |  |  |
| *UBQ* | ACAATGTTAAAGCTAAGATCCA | 392 | 55 |  |
|  | TTAGAAGCCACCACGGAG |  |  |  |
| *SAND* | TCTTGTATCAGGAACCCTGC | 936 | 53 |  |
|  | TCACCATGAAAAAGGGCTTG |  |  |  |
| *SKIP16* | CAATGGATACAACAAACCCAAC | 1340 | 51 |  |
|  | GAAAATGTAATCTGGCAACTGA |  |  |  |
